# Multitasking guardian of mitochondrial quality: Parkin function and Parkinson’s disease

**DOI:** 10.1186/s40035-020-00229-8

**Published:** 2021-01-20

**Authors:** Iryna Kamienieva, Jerzy Duszyński, Joanna Szczepanowska

**Affiliations:** Nencki Institute of Experimental Biology, Polish Academy of Science, 02-093 Warsaw, Poland

**Keywords:** Mitochondria, Parkinson’s disease, Parkin, PINK1, Mitophagy, Parkin mutations

## Abstract

The familial form of Parkinson’s disease (PD) is linked to mutations in specific genes. The mutations in *parkin* are one of the most common causes of early-onset PD. Mitochondrial dysfunction is an emerging active player in the pathology of neurodegenerative diseases, because mitochondria are highly dynamic structures integrated with many cellular functions. Herein, we overview and discuss the role of the *parkin* protein product, Parkin E3 ubiquitin ligase, in the cellular processes related to mitochondrial function, and how *parkin* mutations can result in pathology in vitro and in vivo.

## Introduction

The protein product of *parkin* is a 465-amino-acid E3 ubiquitin ligase [1, 2] that mediates numerous cellular processes upon activation, many of which are tightly connected to mitochondria, an organelle central to cell metabolism, intracellular Ca^2+^ homeostasis, adaptation to stress and cell death [3]. Mitochondrial dysfunction is one of the major hallmarks of Parkinson’s disease (PD) on the cellular level [4], and an insight into how Parkin is implicated in this impairment can reveal the mechanisms of the process.

PD is one of the most common neurodegenerative disorders with cardinal signs of resting tremor, bradykinesia and rigidity, which mostly result from prominent death of dopaminergic (DA) neurons in the substantia nigra (SN) pars compacta [5]. PD is likely to be attributed to various factors, including ageing, genetic susceptibility and environment [4]. Since the disease discovery over 200 years ago, there has been no disease-modifying treatment for PD [5]. Although most of the PD cases are idiopathic (sporadic), i.e., their aetiology is undefined, 5–10% of cases are related to mutations in particular genes and are therefore classified as familial (monogenic) form of PD [6]. Since the discovery of the first causative gene *SNCA* (encoding α-synuclein) in 1997 [7], 19 other genes have been identified [6]. Depending on the mode of inheritance, these genes are categorized into autosomal dominant (e.g., *SNCA*, *LRRK2*, *HTRA2* and *VPS35*) and autosomal recessive (e.g., *PRKN*, *PINK1*, *DJ-1*, *ATP13A2*, *PLA2G6*, *FBXO7*, *DNAJC6*, and *VPS13C*) [6]. For autosomal dominant PD, a single mutated copy of the gene is enough to cause the disease, whereas for autosomal recessive PD, both gene alleles must be affected [8]. One mutated allele of the recessive genes can be a factor for the susceptibility to the development of PD [8]. Understanding the cellular pathways of these genes can help to elucidate the mechanisms of PD pathogenesis and find novel routes for the development of effective treatment.

Mutations in *parkin* (*PRKN* or *PARK2*) are the most frequent cause of autosomal recessive PD [9] and account for 10–20% of early-onset PD (age at onset < 40–50 years) in general [10]. A wide range of *parkin* mutations has been detected, from point mutations to exon rearrangements [11]. Depending on the mutation type and position in the mutant protein, the biochemical consequences of mutations may vary; however, no clear relationship between the nature of the mutation and the clinical severity of the disease has been found [12]. Therefore, in the current review, we will focus on Parkin properties, the main aspects of Parkin function regarding mitochondria, and functional consequences of *parkin* mutations in cellular and animal models.

## Parkin structure and activation

### Parkin structure

Parkin is an E3 ubiquitin ligase that facilitates covalent attachment of ubiquitin from E2 enzymes to a lysine residue of a specific protein substrate [2]. Parkin belongs to the RING-between-RING (RBR) family of ligases, and also has characteristics of two other families, Really-Interesting-New-Gene (RING) and Homologous-to-the-E6-AP-Carboxyl-Terminus (HECT) families [13, 14]. As a RING ligase, Parkin utilizes the canonical RING domain to recruit an E2-conjugating enzyme while as a HECT ligase, Parkin has a catalytic site cysteine that forms an intermediate thioester bond with ubiquitin [13].

**Fig. 1:**
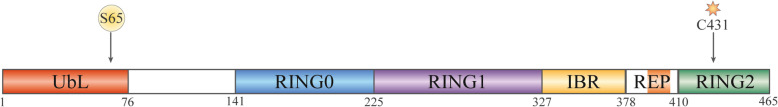
Primary structure of Parkin with colour-coded domains and blank linker sequences

Parkin is comprised of N-terminal ubiquitin-like domain (UbL), RING0 (also known as Unique Parkin Domain, UPD), RING1, In-Between-Domain (IBR), Repressor Element of Parkin (REP), and C-terminal RING2 [15] (Fig. 1). The UbL domain shares approximately 30% sequence identity with ubiquitin [1, 16] and is connected to RING0 through a particularly flexible linker [17]. IBR, RING0, RING1 and RING2 coordinate two Zn^2+^ ions each [13, 18]. The E2-binding site is localized in the RING1 domain, whereas the RING2 domain contains the catalytic cysteine site [13, 18].

### Parkin activation

**Fig. 2:**
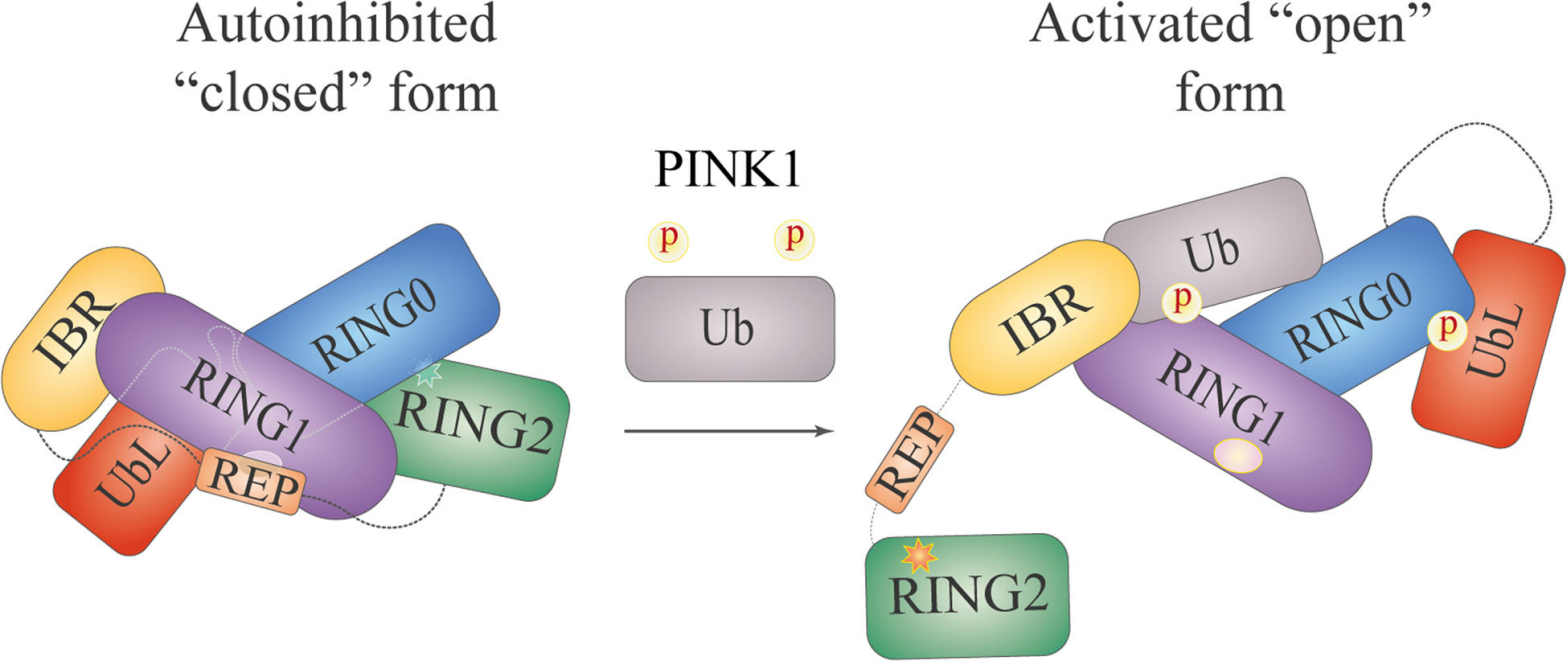
Conformational changes in Parkin upon activation based on the model by Sauvé V. et al. [32]. Star represents the catalytic site and ellipse represents the E2-binding site

Under basal conditions, the cytosolic Parkin exists in an autoinhibited “closed” form [13, 15, 16, 18]. Access to the E2-binding site is blocked by the UbL domain and REP, and RING0 occludes the catalytic site in RING2 [15, 18] (Fig. 2, left part). To gain its enzymatic activity, Parkin must undergo substantial conformational rearrangements. Activation of Parkin is a rather complex process that involves two major events: binding of ubiquitin phosphorylated at serine 65 (pUb^S65^) and phosphorylation of Parkin itself at serine 65 (pParkin^S65^).

Initial insight into the mechanism of Parkin activation was obtained by studies in *Drosophila*, where the protein product of another PD-associated gene, PTEN-induced putative kinase protein 1 (*PINK1*), was found to act upstream of Parkin [19–21]. Later, it was shown that the activated PINK1 can directly phosphorylate Ser65 in the UbL domain of Parkin [22] (Fig. 1), and substitution of Ser65 with alanine (Parkin^S65A^) significantly reduces the ability of Parkin to ubiquitinate its substrates and the rate of Parkin translocation to depolarized mitochondria (discussed later) [23, 24]. Interestingly, PINK1 phosphorylates the isolated UbL domain faster than the full-length protein [25]. Molecular modelling of pParkin^S65^ has also emphasized the importance of PINK1-dependent phosphorylation in the “opening” of Parkin structure [17]. However, a number of observations have indicated that Parkin^S65A^, which cannot be phosphorylated, does not completely lose its enzymatic activity in the cells [24, 26] or the translocation ability [17, 24, 27], and that Ser65 in the autoinhibited Parkin is localized within the core of UbL, which makes it poorly accessible to modifications [22, 28]; these results suggest that an additional step of Parkin activation is required. Extensive studies have led to the discovery of ubiquitin as another PINK1 substrate that is also phosphorylated at Ser65 [24, 29, 30] and interacts with Parkin on the surface of the IBR, RING1 and RING0 domains [28, 31, 32]. The binding of pUb^S65^ induces conformational reorganization of the IBR-REP linker that drives it away from the Parkin core, which in turn leads to UbL dissociation from RING1 [31]. The released UbL can then be efficiently phosphorylated by PINK1 at Ser65. Upon phosphorylation, UbL binds to RING0 with subsequent release of RING2 and REP [32, 33] (Fig. 2, right part). These conformational changes in the Parkin structure result in opening of the E2-binding site in RING1 and the catalytic site in RING2, thus enabling Parkin to bind the E2-ubiquitin conjugate and to transfer ubiquitin to the catalytic cysteine.

Although the narrative of Parkin activation is currently established, a line of evidence indicates that it is not straightforward. Parkin has weak autoubiquitination activity [13] and can be phosphorylated by PINK1 without pUb^S65^ in vitro [22]. Moreover, during ubiquitination in vitro, either Parkin^S65A^ alone or ubiquitin^S65A^ alone negatively impacts the Parkin activity; however, neither can completely abolish this weak activity [26]. In addition, phosphorylation at Ser65 has been shown to substantially enhance Parkin affinity to pUb^S65^ [34]; reciprocally, binding of pUb^S65^ increases the rate of Parkin phosphorylation at Ser65 [35]. These findings suggest that Parkin undergoes dynamic domain movements and that Parkin phosphorylation and pUb^S65^ binding shift the dynamic equilibrium of the Parkin conformation towards its “activated” form [31, 32].

In addition to being phosphorylated at Ser65 by PINK1, Parkin is also a substrate for CDK5 (cyclin-dependent kinase 5) and c-Abl (Abelson tyrosine-protein kinase 1) that phosphorylate Parkin at serine 131 and tyrosine 143, respectively [36, 37]. Both modifications reduce Parkin ubiquitination activity [36–38]. Furthermore, earlier studies have reported an increase in tyrosine-phosphorylated Parkin in cells in response to stress induced by treatment with neurotoxic 1-methyl-4-phenylpyridinium or dopamine [37, 38].

Parkin can also be S-nitrosylated at cysteine 323 [39]. There are conflicting reports on how this modification modulates Parkin activity; some studies claim that it inhibits the autoubiquitination activity of Parkin [40, 41], while others reported that S-nitrosylation activates Parkin and promotes Parkin-mediated aggregation and degradation of mitochondria upon mitochondrial depolarization [39].

Sulfhydration of Parkin has been described to enhance the autoubiquitination activity of Parkin [41].

## Involvement of Parkin in mitochondrial processes

As an E3 ligase, Parkin mediates both multiple monoubiquitination and polyubiquitination of its substrates [42–44]. Parkin can assemble canonical (through lysines K48 and K63) and noncanonical (through K6 and K11) ubiquitin chains [34, 43, 45]. Upon binding to the protein substrates, the K48-linked ubiquitin targets them for proteasomal degradation, while other linkages can modify protein interactions, activity and localization [46].

**Fig. 3:**
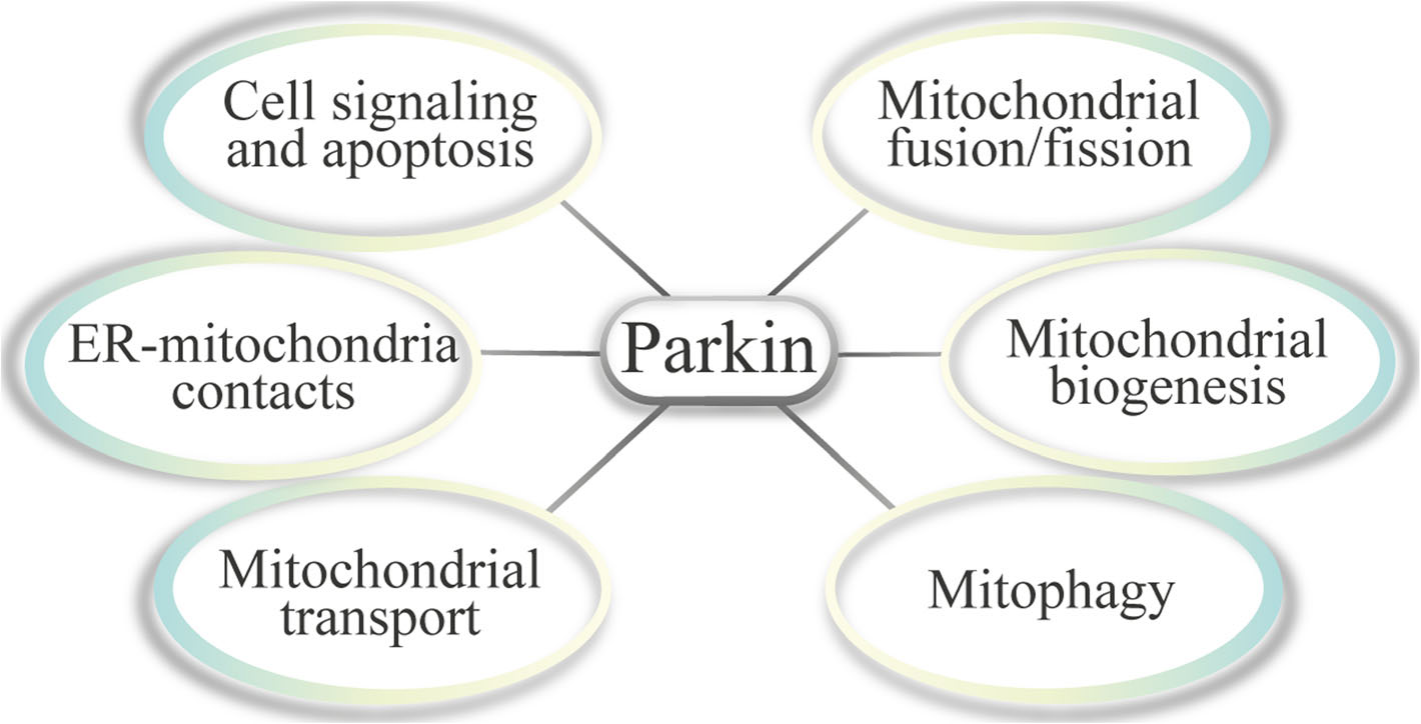
Parkin-mediated cellular processes discussed in this review

Parkin interacts with and promotes ubiquitination of various proteins, mediating a diversity of cellular processes under basal and stress conditions, although no specific target sequence has been found for its recognition of substrates, suggesting that the specificity of Parkin is driven by its proximity to a substrate [47]. This concept is supported by observations that exogenous proteins targeted to mitochondria are ubiquitinated by activated Parkin [48] and when Parkin is recruited to peroxisomes by ectopic PINK1, it can efficiently drive ubiquitination of their surfaces [49]. Particularly, Parkin activity is tightly connected to diverse aspects of mitochondrial functioning, and some of these processes will be reviewed in the following subsections (Fig. 3).

### Mitochondrial fusion/fission

Mitochondria form a dynamic network maintained by the antagonistic processes of fusion and fission, which reflect metabolic demand of the cell. Therefore, the regulation of fusion/fission events is essential for the maintenance of proper cell functions, especially in response to the changing physiological conditions or cellular stressors.

Early studies of mutant *Drosophila* with loss-of-function of *parkin* have revealed mitochondrial pathology manifested as mitochondrial elongation [50, 51], swelling [50, 52, 53] and cristae disruption [50, 52–54]. Mitochondria are more fussed in dopaminergic (DA) neurons of the protocerebral posterior lateral 1 (PPL1) cluster in the *parkin* mutant of *Drosophila* [55]. In contrast, a study has reported mitochondrial fragmentation and swelling in PPL1 DA neurons [56]. The frequently observed mitochondrial phenotype of the *parkin*-mutant *Drosophila* can be markedly rescued by overexpression of a pro-fission protein dynamin-1-like protein (Drp1) or by knockdown of pro-fusion protein homologue of mitofusins (Marf) or optic atrophy protein 1 (Opa1), which suggest that Parkin promotes fission and/or inhibits mitochondrial fusion [50, 53, 57]. However, studies in mammalian cell lines have introduced a discrepancy into the picture. Silencing of the *parkin* gene in human neuroblastoma cell line SH-SY5Y [58–61] resulted in fragmentation of mitochondria. Accordingly, overexpression of Mfn2, Opa1 or dominant-negative Drp1 reversed this mitochondrial alteration [59]. On the contrary, other studies in simian kidney fibroblast cell line (COS7) [62] and rat hippocampal and dopaminergic neurons [63] reported that Parkin overexpression rather than knockdown caused mitochondrial fragmentation. The discrepancy on the effects of Parkin on mitochondrial fusion/fission may be due to the differences in the constructs used to introduce Parkin alterations or to the tissue-specific activity of Parkin.

Nevertheless, the fact that Parkin can influence the fusion/fission of mitochondria is undeniable, especially when considering its influence on the protein elements of the fusion/fission machinery. Upon activation, Parkin ubiquitinates various proteins of the mitochondrial outer membrane [47]. Particularly, both mitofusins (Mfn1/2) have been identified as the direct Parkin substrates [47, 51, 64–68]. Parkin knockdown does not influence the expression of mitofusins [58, 67]; however, upon mitochondrial depolarization, the Parkin-dependent ubiquitination [64] promotes proteasomal degradation of mitofusins [66, 67]. Parkin can also mediate the upregulation of Opa1, which is involved in the fusion of inner mitochondrial membrane and maintenance of the cristae structure, in response to certain stress conditions, and silencing of the *parkin* gene leads to a decrease in the expression of Opa1 [60]. Expression of Drp1, the main player in mitochondrial fission, appears to be enhanced upon Parkin knockdown [58], similar to the expression of one of the Drp1 recruitment partners, Mff [69]. Interestingly, Drp1 could not directly interact with Parkin [67], whereas Mff could [70]. Notably, the Parkin-dependent ubiquitination leads to degradation of both proteins via different pathways; Drp1 undergoes proteasomal degradation [58], while Mff is degraded via the lysosomal route [69]. Another Drp1 recruitment factor, Fis1, has also been described as a protein that directly interacts with Parkin [47].

### Mitochondrial biogenesis

Mitochondrial biogenesis is regulated by numerous signalling pathways, resulting in a new pool of mitochondria within the cell.

The role of Parkin in mitochondrial biogenesis is primarily mediated by its influence on peroxisome-proliferator-activated receptor gamma coactivator 1-alpha (PGC1-α), one of the master regulators of mitochondrial function and biogenesis. The PGC1-α repressor, PARIS, is ubiquitinated by Parkin, which subsequently leads to its proteasomal degradation [71]. Interestingly, the PINK1-mediated phosphorylation of PARIS facilitates its ubiquitination by Parkin [72]. Conditional Parkin knockout mice exhibit a reduction in the number and size of mitochondria in the ventral midbrain neurons, and these changes can be rescued by PARIS knockdown [73]. Overexpression of Parkin increases the relative copy number of mitochondrial DNA [74], and loss of Parkin results in a decrease in the mitochondrial DNA copy number, which can be restored by PARIS knockdown [73].

In addition, Parkin deficiency can affect cell bioenergetics as it results in the Warburg effect, enhancing glucose uptake, the rate of glycolysis, and lactate production [75] while reducing oxygen consumption, which in turn decreases mitochondrial respiration [73–75].

### Mitophagy

Since the finding of similar phenotypes caused by loss of Parkin or PINK1 and that Parkin and PINK1 participate in the same pathway [19–21], considerable number of studies has been done to understand the extent of this interaction. The results of extensive investigations have linked both proteins in the process of PINK1/Parkin-mediated mitophagy. This particular type of selective autophagy is designated to eliminate dysfunctional mitochondria and can be triggered by depolarization of the mitochondrial membrane (experimentally induced with Δψ-dissipating agents, e.g., H^+^ ionophores CCCP [carbonyl cyanide 3-chlorophenylhydrazone] or FCCP [carbonyl cyanide 4-trifluoromethoxyphenylhydrazone], K^+^ ionophore valinomycin, or a combination of a complex III inhibitor antimycin A and an ATP synthase inhibitor oligomycin) [22, 76, 77], reactive oxygen species (ROS) [78, 79], accumulation of misfolded proteins in the mitochondrial matrix [80], paraquat [77, 81] or NO [39, 82].

In PINK1/Parkin-mediated mitophagy, PINK1 serves as a sensor of mitochondrial damage and is readily degraded under the basal conditions. This serine-threonine kinase contains a mitochondria-targeting sequence (MTS) at the N-terminus which is imported across the mitochondrial outer (MOM) and inner (MIM) membranes via TOM and TIMM23 complexes, respectively [83, 84]. Then, the MTS of PINK1 is removed by mitochondrial processing protease, and PARL (intramembrane presenilin-associated rhomboid-like protein) of MIM cleaves PINK1 within its transmembrane domain [85]. The cleaved PINK1 is released into the cytosol where it undergoes degradation by proteasome via the N-end rule pathway [81, 83]. However, upon mitochondrial depolarization, PINK1 translocation through the MIM is inhibited and PINK1 is thus stabilized on the mitochondria [81, 85, 86]. Accumulation of PINK1 on the membranes together with its kinase activity is crucial for Parkin translocation and activation, as artificially targeting PINK1 to the mitochondrial, ectopic peroxisomal, or lysosomal membranes could recruit Parkin, leading to ubiquitination of the membrane proteins [49, 81], and the kinase-dead PINK1 is unable to recruit Parkin [49, 86].

**Fig. 4:**
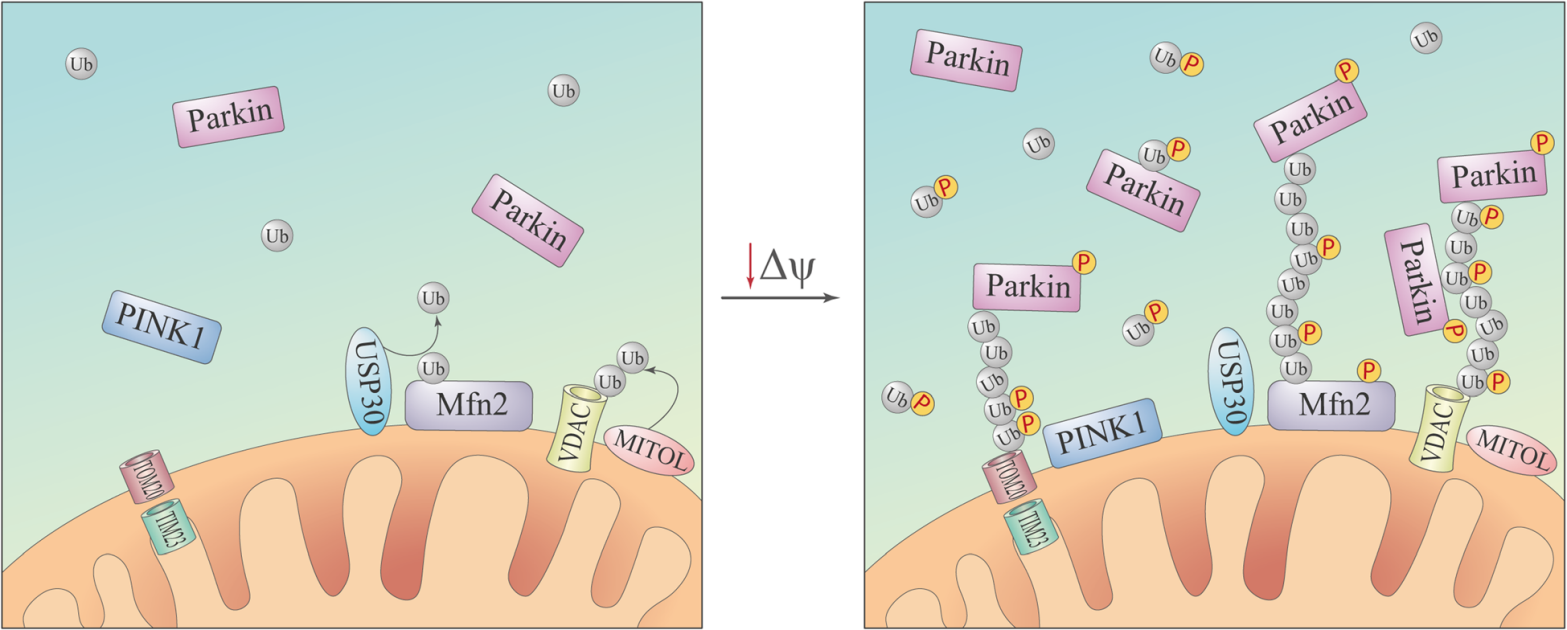
Model of the PINK1/Parkin-mediated polyubiquitination of MOM upon mitochondrial depolarization. Under basal conditions, ubiquitination in mitochondria is balanced by resident ubiquitin E3 ligases (e.g., MITOL) and deubiquitination enzymes (e.g., USP30) (left panel). However, upon mitochondrial depolarization, stabilized PINK1 promotes the Parkin-dependent generation of ubiquitin chains on MOM (right panel)

Parkin translocation from the cytosol to the mitochondria occurs in two main stages. Mitochondria are moderately ubiquitinated under basal conditions (e.g., by resident mitochondrial ubiquitin E3 ligases such as mitochondrial ubiquitin ligase [MITOL]) [48] (Fig. 4, left panel). This ubiquitination is kept at a low level by the deubiquitination enzymes [45]. Notably, USP30 (ubiquitin-specific-processing protease 30) was identified as a regulator of PINK1/Parkin-mediated mitophagy [45, 87]. Parkin was proposed to be continuously sampling the mitochondria surface by a diffusion-controlled process [34]. Upon decrease of Δψ, the stabilized PINK1 phosphorylates ubiquitin and Parkin in a close proximity [24]. On the initial stage, binding of pUb^S65^ to Parkin [24, 26, 29] and its phosphorylation at Ser65 by PINK1 [22, 23] result in Parkin release from the autoinhibited state, inducing subsequent polyubiquitination of the Parkin protein substrates on MOM. On the next stage, newly formed ubiquitin chains are phosphorylated by PINK1 [30, 88]; Parkin also can assemble phosphorylated ubiquitin chains [34]. The phosphorylated ubiquitin chains physically interact with Parkin [30, 34] and facilitate docking of activated Parkin and its retention at the mitochondria surface, which then results in the synthesis of new ubiquitin chains and the amplification of the signal [89] (Fig. 4, right panel). Interestingly, the introduction of phosphomimetic Parkin mutants and phosphomimetic polyubiquitin chains promotes translocation of the former to energized mitochondria even without the expression of PINK1 [88]. Proper E3 ligase activity of Parkin is required for this stage, because catalytically inactive Parkin (Parkin^C431S^) does not translocate to mitochondria after CCCP treatment [17, 34, 43, 88–90], but its co-expression with wild-type Parkin or mitochondria-targeted ubiquitin chains facilitates Parkin^C431S^ recruitment to the mitochondria [30, 89].

Activated Parkin ubiquitinates a variety of MOM proteins [47]. The chains of ubiquitin are recognized by the mitophagy receptors [91], which eventually leads to engulfment of the impaired mitochondria in a bit-by-bit manner into autophagosome [78] and their subsequent elimination in the lysosome. The Parkin-dependent ubiquitination of myosin VI (MYO6) is particularly important for mitophagy, since MYO6 induces the formation of F-actin cages around the damaged mitochondrial fragments, preventing their fusion with the rest of the mitochondrial network [92].

The mechanism of PINK1/Parkin-mediated mitophagy has been well characterized in a number of cell lines; however, the relevance of the mechanism to basal conditions/moderate stress or in cells that predominantly rely on oxidative phosphorylation (e.g., neurons) remains rather undefined. In a comparative study, no mitochondrial translocation of Parkin was observed upon treatment with CCCP in rat cortical neurons and in galactose-cultured HeLa cells (galactose forces the cells to rely on mitochondrial ATP production) after 1 or 3 h [93]. In another study, Parkin recruitment and mitophagy were detected as early as after 12 h of treatment with Δψ-dissipating agents in mouse cortical neurons, suggesting that the PINK1/parkin-mediated mitophagy in neurons is a considerably slower process than that in the non-neuronal cells [94]. Moreover, Parkin knockdown in the neurons disrupted the elimination of dysfunctional mitochondria [87, 94]. Local PINK1/Parkin-mediated mitophagy was detected in the axons of rat hippocampal neurons within 1 h of local induction [79]. Thus, mitophagy is a highly orchestrated process that may lead to various outcomes depending on the type, strength and duration of a stress and on the cell type.

### Mitochondrial transport

The transport of mitochondria to cellular areas with high energy demand is particularly important for polarized cells, e.g., neurons.

**Fig. 5:**
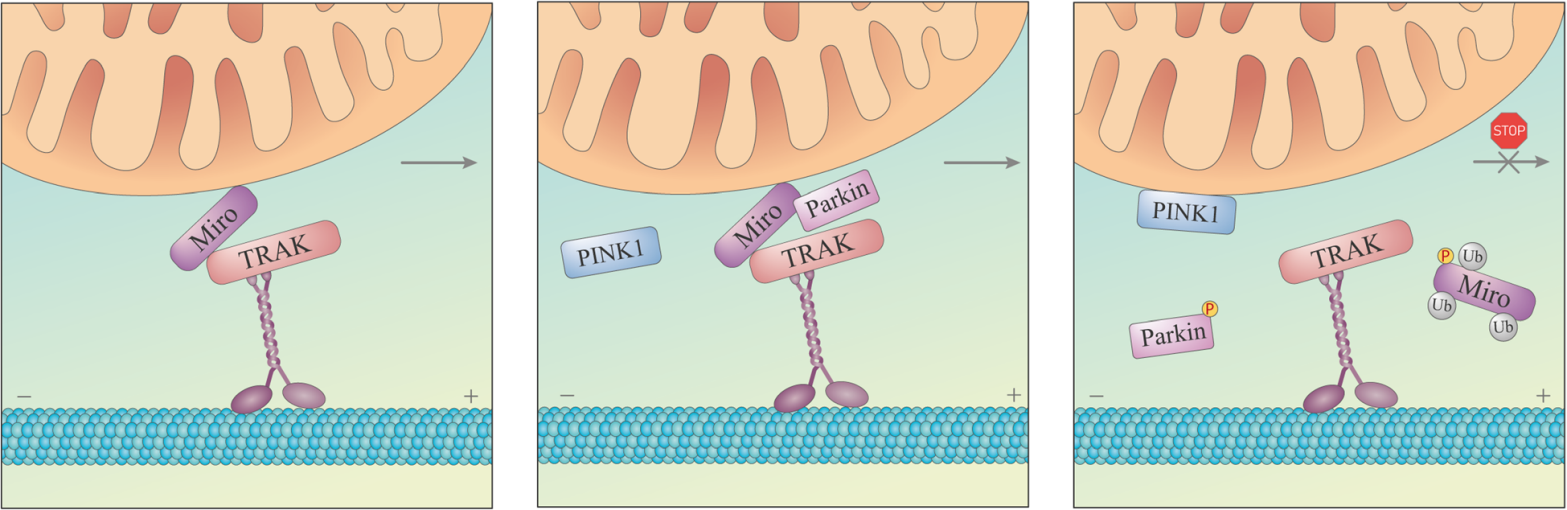
Model of the PINK1/Parkin-mediated arrest of mitochondrial motility upon mitochondrial depolarization. Mitochondria are attached to the motor protein kinesin on the microtubules *via* adaptors TRAK and Miro with Miro anchored in MOM (left panel). Parkin possibly interacts with Miro before mitochondria are damaged (middle panel). After stabilization of PINK1 on MOM, activated Parkin catalyses monoubiquitination of Miro at multiple sites (right panel), resulting in dissociation of the mitochondria from the microtubules

Parkin is implicated in mitochondrial transport via ubiquitination of the mitochondrial Rho GTPase (Miro) proteins (Miro1/2). Miro is a part of the motor adaptor complex that connects mitochondria to the microtubules. Miro, trafficking kinesin-binding protein (TRAK) and kinesin heavy chain complex of kinesin-1 motor proteins (collectively known as KIF5) comprise one of the most well studied anterograde transport systems [95]. Both Miro1 and Miro2 were established as direct substrates of Parkin [47]. Miro1 and Miro2 are multi-monoubiquitinated by Parkin, although Miro1 is modified more robustly than Miro2 [96]. Parkin co-precipitates with Miro1 even under basal conditions, and this interaction is enhanced upon Parkin overexpression or mitochondrial depolarization [97, 98]. Ultimately, overexpression of Parkin or depolarization of the mitochondria results in proteasomal degradation of Miro [27, 98] that leads to dissociation of the motor adaptor complex from the mitochondrial surface, eventually resulting in arrest of mitochondrial motility [98] (Fig. 5). In axons in particular, Parkin translocation to the mitochondria induces a decrease in the anterograde transport and a comparative increase in the retrograde transport accompanied by reduced velocity of axonal mitochondria, which may restrict mitochondrial trafficking to the cell periphery [94]. Phosphorylation of Miro1 at S156 by PINK1 was found to facilitate Parkin-dependent degradation of Miro1 upon mitochondrial depolarization [98, 99]. Mimicking this modification with substitution for glutamic acid (S156E) enhanced mitochondrial fragmentation and decreased mitochondrial motility [99]. Interestingly, overexpression of Miro1 [97, 99] or its phosphomimetic mutant S156E [99] promoted Parkin translocation to the mitochondria independently of PINK1 or the status of Δψ. However, other studies failed to observe any major influence of phosphorylation at S156 on Miro1 degradation [27, 100].

In addition, Parkin overexpression has been found to decrease the protein level of TRAK1 in a proteasome-dependent manner [98].

### Endoplasmic reticulum (ER)–mitochondrial contacts

**Fig. 6:**
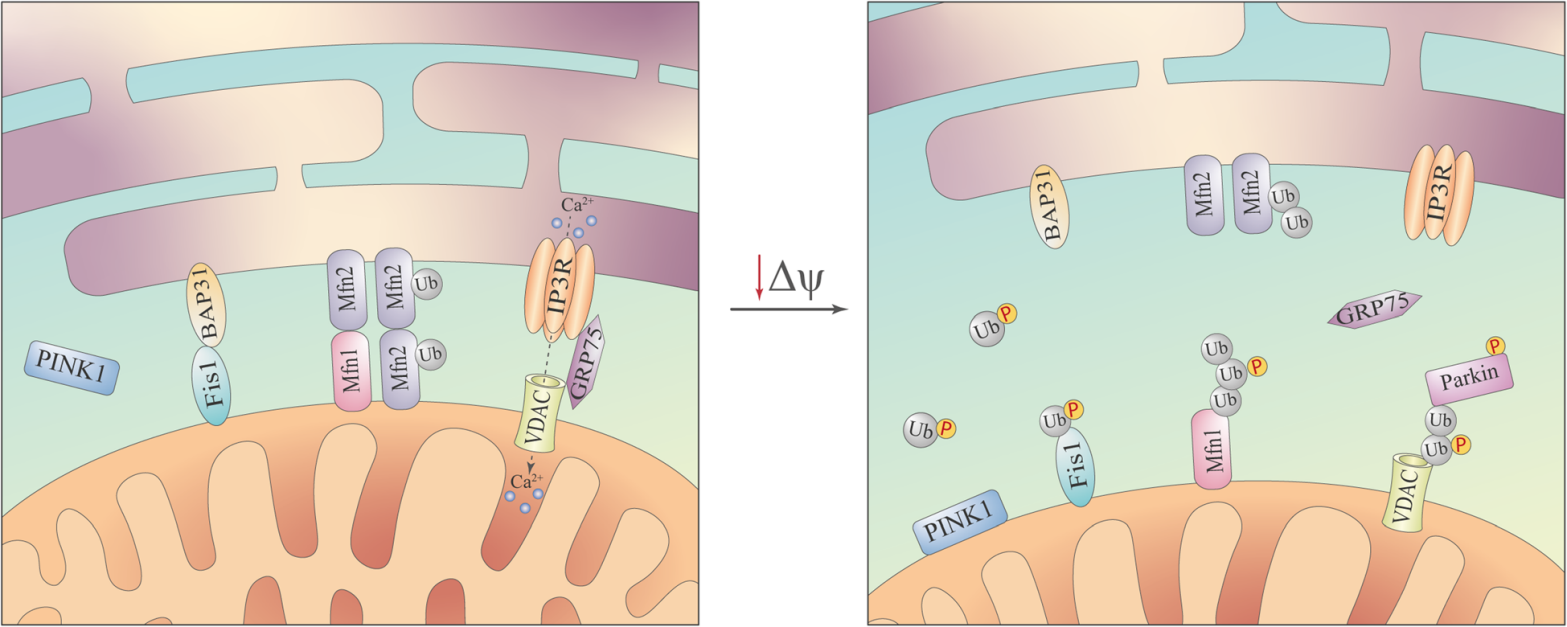
Model of disintegration of the ER–mitochondrial contact sites upon mitochondrial depolarization. Under basal conditions, the contacts between the ER and mitochondria are mediated by various proteins, including Mfn2, ubiquitination of which at K416 was suggested to promote the interaction (left panel). Upon mitochondrial depolarization, Mfn2 is polyubiquitinated and is removed from the membrane for proteasomal degradation, resulting in disruption of the ER–mitochondria contact sites and drifting of these organelles away from each other (right panel)

Parkin can regulate the ER–mitochondrial interactions. The contact sites between the mitochondria and ER synergize the functions of the two organelles and facilitate numerous signalling pathways in the cell. Overexpression of Parkin increases the co-localization of ER with mitochondria and enhances agonist-stimulated Ca^2+^ mitochondrial influx [61]. Likewise, Parkin downregulation reduces the ER–mitochondria tethering [101] and negatively affects mitochondrial Ca^2+^ uptake [61]. This effect may be mediated by the regulatory Parkin-dependent ubiquitination of Mfn2 at K416 [101], because Mfn2 is a component important for the formation of the ER–mitochondrial contact sites [102] (Fig. 6, left panel). Moreover, ubiquitination and subsequent degradation of Mfn2 facilitate disintegration of the ER–mitochondrial contact sites during mitophagy [103] (Fig. 6, right panel). This mechanism is supported by preferential ubiquitination of damaged mitochondria at the ER–mitochondrial contact sites [78].

### Cell signalling and apoptosis

Given that mitochondria are at the intersection of various metabolic and signalling pathways, the impact of this organelle on cell function and cell destiny is undeniable.

Parkin has been found to be cytoprotective under various cellular stress conditions, indicating its participation in the cell survival signalling pathways [60, 104–106]. Loss of Parkin sensitizes the cells towards stress-induced cell death [60], and certain cellular stressors can boost Parkin expression [60, 75]. These effects of Parkin are partially achieved by ubiquitination of pro-apoptotic factor Bax that prevents Bax translocation to the mitochondria [105] and subsequent cytochrome *c* release [104]. Moreover, there are reports of Parkin interaction with p53. Putative p53-responsive elements have been detected in the *parkin* gene, and p53 activation increases Parkin expression [75]. Likewise, Parkin has been claimed as a transcriptional repressor of p53 that directly binds to the p53 promoter [106]. This interaction is plausible because the RING0 domain of Parkin is similar to the Zn-finger domains [13], and certain studies have reported nuclear localization of Parkin [106, 107]; however, the exact mechanism of Parkin translocation to the nucleus is unclear. Although Parkin interaction with p53 has been verified, the p53–Parkin transcriptional regulation remains to be confirmed [108].

Parkin has also been shown to facilitate the activation of the pro-survival NF-κB pathway under the stress conditions by promoting the ubiquitination of NF-kappa-B essential modulator (NEMO) [60] and regulatory IκB kinase IKKγ [109]. Moreover, the ubiquitination of mammalian target of rapamycin (mTOR) kinase by Parkin appears to be important for the maintenance of the mTOR complex 1 activity in response to mitochondrial stress [110].

Apart from the functions described above, Parkin is also implicated in cell cycle regulation [111–113], vesicular trafficking [114–116], repression of mitochondrial antigen presentation [117] and synaptic functioning (reviewed in [118]), which makes Parkin an important cellular regulator.

## Parkin and PD

The *parkin* gene was discovered in search for causative genes of what appeared to be familial autosomal recessive PD in a particular Japanese family [1]. Subsequent identification of this gene and its corresponding protein product made *parkin* the second most common gene associated with familial PD after *PARK1* (*SNCA*, α-synuclein) [7], and therefore, was named *PARK2* [1].

Although clinical features of PD are similar between PD patients with and without *parkin* mutations, the mutation carriers are characterized by slow progression, early onset (21–50 years), good response to levodopa treatment, and prevalence of dystonia, dyskinesia and diurnal fluctuations [11, 119]. With regard to the non-motor signs, dementia is rarely found in PD patients with mutations [11]. Typically, Lewy bodies (distinctive protein aggregates found in brains of PD patients) are not detected in PD patients with *parkin* mutations during autopsy [120].

Postmortem analysis of brain tissues of patients with sporadic PD has revealed a range of changes related to the Parkin protein. Expression of Parkin is reduced in the striatum, but not in the cortex, of PD patients compared to that in the controls. Moreover, the decreased Parkin solubility in the striatum of PD patients has been detected along with increased levels of ubiquitinated proteins [121]. Elevated levels of tyrosine-phosphorylated Parkin have been observed in the postmortem striatum and SN of PD patients [37, 38]. An increase in parkin S-nitrosylation and a decrease in its sulfhydration have been found in the postmortem brain tissues from PD patients [40, 41]. In addition, upregulation of the Parkin substrate, PARIS, has been detected in the striatum and SN of PD patients [71].

### Diversity of parkin mutations in PD

According to the NCBI database, the *parkin* gene is approximately 1.38 Mb long, which makes it one of the largest genes in the human genome; the gene contains 12 exons [1]. The *parkin* gene is widely expressed in human tissues, including the brain, heart, testis and skeletal muscle [1]. Importantly, *parkin* is located in 6q26 within the FRA6E common fragile site [122], which may explain why *parkin* is one of the most frequently deleted genes in human cancer [113].

*Parkin* mutations are found in heterozygous (only one allele affected), compound heterozygous (both alleles affected by different mutations) or homozygous (both alleles affected by the same mutation) states [123, 124]. Individuals with two mutated *parkin* alleles have earlier age of disease onset and age of disease diagnosis compared to those having only a single mutated *parkin* allele [123–125]. Heterozygous *parkin* mutations have also been detected in clinically unaffected healthy individuals [126]. Moreover, studies on asymptomatic carriers of heterozygous *parkin* mutations have revealed signs of nigrostriatal dysfunction [127], alterations in movement-related activation patterns in the brain [128] and impaired facial emotion recognition [129].

**Fig. 7:**
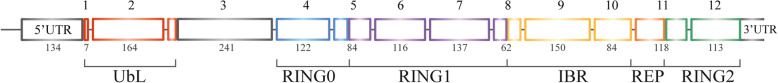
Exon structure of the *parkin* gene with colour-coded corresponding protein domains based on the data of Fiesel et al. [137]

*Parkin* mutations may occur in all the 12 exons (Fig. 7) and vary from single-nucleotide changes and small deletions to rearrangements of a single or several exons with copy number variations (deletions and duplications) [11]. Deletions, duplications and nonsense point mutations result in a protein product lacking a certain part (in-frame mutations) or a truncated protein (out-of-frame mutations) [123]. For example, deletion of exon 3 generates the truncated 76-amino-acid Parkin, whereas deletion of both exons 3 and 4 results in Parkin lacking a 58–176 amino-acid sequence [123]. Moreover, dosage mutations of *parkin* appear to have greater pathogenicity than single sequence mutations [125].

To date, there has been a record of 479 *parkin* mutations in the Human Gene Mutation Database (HGMD, http://www.hgmd.cf.ac.uk/ac/index.php) [130], and approximately 400 mutations are related to PD, with approximately 350 mutations being reported to be PD disease-causing. Of note, almost half of the mutations in the database are “gross deletions” and may thus be overestimated (similar to the “gross insertions” section), since each mutation is logged in a narrative form because of the extremely variable quality of the originally reported data. Systemic reviews account for various numbers of *parkin* mutations in PD probably due to the specific inclusion criteria and study limitations: 183 mutations [11] or 139 disease-causing sequence variants according to a more recent study [119]. The majority of the detected mutations are localized between exons 3 and 8, spanning the centre of FRA6E [122]. Most of the mutations are represented by point mutations, while exon rearrangements are the most frequent mutation types [11]. Among exon rearrangements, deletions of exons 3, 4, or both are the most common, whereas the missense mutation R274W is the most frequent point mutation [11]. However, the pathogenic nature and relevance to the disease are not clear for the majority of these variants.

**Fig. 8:**
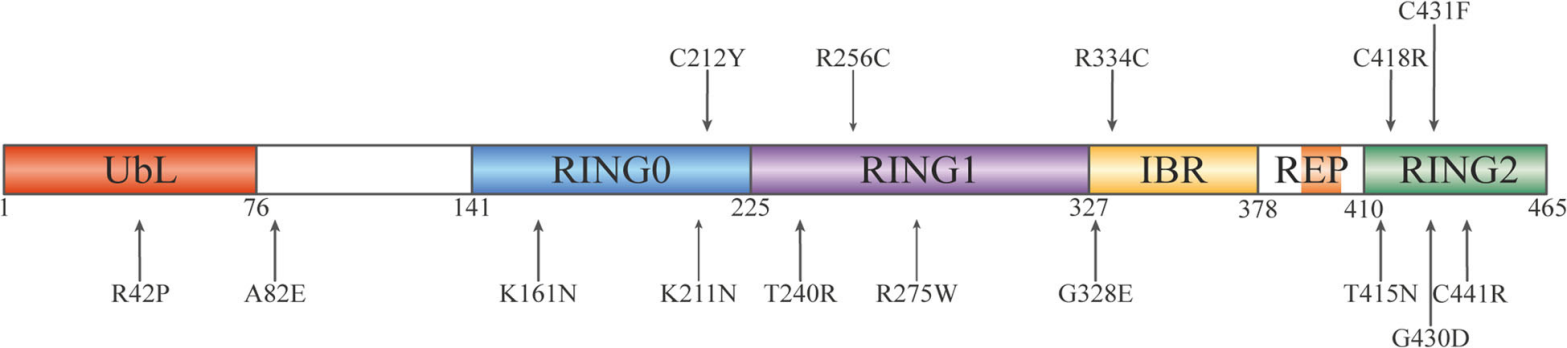
Localization of the most studied point mutations in the Parkin protein sequence

Point mutations of *parkin* can introduce conformational (perturbations of zinc coordination or protein folding) and site changes (disruption of the catalytic site, E2-binding site or substrate-binding sites) into the Parkin protein structure [18]. As a result, the mutations can affect Parkin in the following facets: (1) solubility and intracellular localization [107, 131–133]; (2) autoubiquitination [18, 44, 131, 134] and the ability to ubiquitinate substrates, e.g., CISD1 [34], Mfn1 [66, 67], Mfn2 [34], Miro1 [42], PARIS [71], VPS35 [116], p38 [131] and synphilin-1 [131]; (3) the ability to interact with E2 [2] and the protein substrates [71]; and (4) the ability to translocate to depolarized mitochondria [23, 34, 81, 135–137] and promote mitophagy [81, 135, 136]. Based on the clinical records, *parkin* point mutations have been assigned into five groups by Sherloc (semi-quantitative, hierarchical evidence-based rules for locus interpretation) variant classification: “pathogenic” and “likely pathogenic” (mutations with clear or very likely disease-causing effect), “likely benign” and “benign” (not disease-causing mutations), and “uncertain significance” (variants that cannot be assigned to one of the other four groups) [138]. A brief summary of the most studied *parkin* point mutations is given in Table 1; localization of these mutations within the Parkin protein sequence is indicated by arrows in Fig. 8.

**Table 1:** Examples of *parkin* point mutations detected in Parkinson’s disease patients and their impacts on the structure and activity of the Parkin protein

Mutation	Assignment based on clinical evidence using Sherloc	Biochemical and functional consequences compared to wild-type Parkin
R42P	Pathogenic	Similar solubility [131] vs lower solubility [132, 134] Increased solvent accessibility [139] Formation of intracellular aggregates [131] vs similar diffuse distribution [134] Retained autoubiquitination activity [131, 134] Reduced stability [137, 138, 140] Drastic changes in structure [139] that disrupt the autoinhibition state [16] Translocating to mitochondria [23, 135, 137] vs no translocation to depolarized mitochondria [136] Reduced mitophagy [136, 138] vs normal mitophagy [135] Reduced phosphorylation by PINK1 [25]
A82E	Benign	Similar solubility [132, 134] Similar diffuse distribution [107, 132, 134] Retained autoubiquitination activity [134]
K161N	Uncertain significance	Similar solubility [131, 134] vs lower solubility [132] Similar diffuse distribution [131] Abolished [131] vs reduced [18] vs retained autoubiquitination activity [134] Reduced ubiquitin chain synthesis [34] Loss of charge in the putative phospho-binding site [137] Translocation [135, 137] vs reduced translocation to depolarized mitochondria [23, 34, 86] Reduced mitophagy [135]
K211N	Pathogenic	Similar solubility [132, 134] Similar diffuse distribution [132] Loss of charge in the putative phospho-binding site [137] Retained autoubiquitination activity [134] Reduced ubiquitin chain synthesis [34] Reduced translocation to depolarized mitochondria [23, 34, 81, 86, 135, 137] Reduced mitophagy [138]
C212Y	Pathogenic	Lower solubility [132] Formation of intracellular aggregates [135, 138] Suggested decreased protein stability [138] Reduced translocation to depolarized mitochondria [81] Reduced mitophagy [138]
T240R	Likely pathogenic	Similar solubility [131, 132] Similar diffuse distribution [131, 132] Abolished autoubiquitination activity [18, 131] Significantly affected RING1-UBL binding [137] that affects E2 binding [2] Reduced translocation to depolarized mitochondria [23, 34, 135–137] Induce formation of mitochondrial aggregates [136] Reduced mitophagy [136, 138]
R256C	Uncertain significance	Formation of intracellular aggregates [107, 131] vs similar diffuse distribution [134] Lower solubility [131, 132] vs similar solubility [134] Retained autoubiquitination activity [131, 134] Minor structural variation [137] Translocate to depolarized mitochondria and promote mitophagy [135]
R275W	Likely pathogenic	Lower solubility [131, 132, 134] Formation of intracellular aggregates [107, 131, 132, 134, 138] Retained autoubiquitination activity [131, 134] Fail to promote ubiquitin chain synthesis [34] Disrupt charge distribution and local rearrangements in the RING1-IBR interface [137] Suggested decreased protein stability [138] Translocate to depolarized mitochondria [135–137] Reduced mitophagy [135, 136, 138]
G328E	Uncertain significance	Similar solubility [131, 134] vs lower solubility [132] Similar distribution [107, 131, 134] Retained activity [131, 134] vs reduced autoubiquitination activity [18] Loss of flexibility of the loop region and disturbances of backbone arrangement [137] Translocate to depolarized mitochondria and promote mitophagy [86, 135]
R334C	Benign	Lower solubility [132] No effect on domain stability [137] Translocate to depolarized mitochondria and promote mitophagy [135] vs increase mitophagy [138]
T415N	Pathogenic	Similar solubility [131, 132] Similar distribution [131, 132] Abolished autoubiquitination activity [44, 86, 131] Disturbance of backbone arrangement [137] Reduced translocation to depolarized mitochondria [23, 136, 137] Induce formation of mitochondrial aggregates [136] Reduced mitophagy [136, 138]
C418R	Uncertain significance	Lower solubility [134] Formation of intracellular aggregates [134, 135] Increased solvent accessibility [133] Abolished autoubiquitination activity [134]
G430D	Likely pathogenic	Lower solubility [131] vs similar solubility [132] Similar diffuse distribution [131, 132] Retained autoubiquitination activity [131] vs abolished autoubiquitination activity [44, 86] Reduced ubiquitin chain synthesis [34] Reduced translocation to depolarized mitochondria [23, 34, 135, 137] Reduced mitophagy [138]
C431F	Pathogenic	Lower solubility [131, 132] Similar diffuse distribution [107] vs forming intracellular aggregates [131] Retained autoubiquitination activity [131] vs abolished autoubiquitination activity [44] High local destabilization and loss of the catalytic centre [137] Reduced translocation to depolarized mitochondria [137] Reduced mitophagy [138]
C441R	Pathogenic	Lower solubility [132, 134] Formation of intracellular aggregates [132, 134, 135, 138] Increased solvent accessibility [133] Abolished autoubiquitination activity [44, 134] Do not translocate to depolarized mitochondria [81] Suggested decreased protein stability [138]

### Parkin deficiency in fibroblasts derived from PD patients

Most of the studies on *parkin* mutations have been done in an exclusive genetic context thus excluding the impact of possible environmental cues that accompany the development of PD. Primary skin fibroblasts derived from PD patients represent a research tool for investigation of numerous aspects of PD pathology, since these cells not only retain the genotype of the patient but also present with the same cumulative cellular damage acquired through the patient’s lifetime [141]. Furthermore, the fibroblasts can be reprogrammed into induced pluripotent stem cells and then redifferentiated into another cell type, e.g., DA neurons [141].

Studies on the primary fibroblasts from PD patients with *parkin* mutations are vastly focused on the mitochondria in search for clues to mitochondrial dysfunction observed in PD. Various alterations in the mitochondrial functions have been reported, including changes in mitochondrial respiration, especially after substitution of glucose with galactose in the media that forces the cells to rely on mitochondrial ATP production [142] (Table 2). In addition, the mitochondria appear to have a basal defect in functional connectivity that can be enhanced by rotenone [143]. Surprisingly, a majority of the studies did not find significant changes in the mitochondrial network morphology [68, 142, 144–146] based on the evaluation of form factor (as a measure of mitochondrial branching) and aspect ratio (as a measure of mitochondrial elongation), although some abnormal “chain-like” mitochondria were observed [146], and ultrastructural analysis in some fibroblasts of the patients revealed severe mitochondrial pathology manifested as swollen mitochondria with few remaining cristae and decreased electron matrix density [147]. To our knowledge, there have been no studies on mitophagy in these fibroblasts; however, ubiquitination and degradation of Mfn2 [68] and degradation of Miro1/2 [27] are impaired upon mitochondrial depolarization.

**Table 2:** Conflicting results regarding mitochondria reported in primary fibroblasts from PD patients with *parkin* mutations

Parameter	Reported observations
**Δψ**	Lower Δψ [143, 146] No differences in Δψ [142, 148]
**Complex I activity**	Decreased complex I activity [143, 147] No changes in complex I activity [144]
**ATP production**	Reduced cellular ATP levels [143, 145, 146] Higher cellular ATP levels with increased glycolysis [147]
**Mitochondrial morphology**	Higher form factor [143] Lower form factor [148] No changes in form factor [68, 142, 144, 145] Unaffected mitochondrial aspect ratio [142, 145, 148]
**Mitochondrial mass**	Increased mitochondrial mass [144] Unaffected mitochondrial mass [142, 145]
**Mitochondrial respiration**	Increased basal oxygen consumption [146, 148] Lower basal oxygen consumption [147] Reduced uncoupled respiration rates [147] Decreased basal/maximal respiratory ratio in galactose media [142]

RNA sequencing in fibroblasts from PD patients with *parkin* mutations demonstrated alterations in the expression of genes related to cell adhesion, cell growth, cell motility, glycine and serine amino acid metabolism, and folate metabolism [149]. Changes in protein expression have also been reported, mainly including cytoskeletal structural proteins, stress response proteins, calcium-related proteins, redox balance-related proteins, protein processing-related proteins and RNA processing-related proteins [150].

Moreover, an increase in ROS production [147], higher levels of protein oxidation [144] and lower activity of the antioxidant enzymes (MnSOD, GPx and catalase) [147] have been observed in addition to higher levels of cytosolic and mitochondrial Ca^2+^ [151].

In DA neurons derived from induced pluripotent stem cells generated from the dermal fibroblasts of PD patients with *parkin* mutations, spontaneous DA release, diminished specific DA uptake and reduced amount of DAT binding sites have been detected [152]. These neurons also exhibit oscillatory neuronal activities [153] resembling those detected in the basal ganglia neurons of PD patients [154]. Moreover, these DA neurons display reduced complexity of neuronal processes compared to the neurons derived from control fibroblasts [155]. Mitophagy appears to be unaffected [152]. Notably, the efficacy of differentiation into DA neurons is reduced in the patient lines [156]. Elevated levels of α-synuclein without formation of its aggregates have also been reported [156].

Mitochondria in the cell bodies of DA neurons derived from fibroblasts with *parkin* knockout exhibit signs of swelling and irregular, dilated cristae [156]; mitochondrial elongation was observed in another study [157]. The gene expression profile of these cells is altered, including several mitophagy-related genes [156]. Mitochondria-specific proteomics has detected differentially regulated proteins related to oxidative stress defence, energy metabolism, and cell cycle regulation [157]. Neurons with *parkin* knockout exhibit reduced efficiency of reprogramming into DA neurons and a decrease in the total neurite length [158].

### Parkin deficiency in animal models

To better understand the impact that Parkin deficiency has in an organism, considerable amount of work has been done in animal models, mostly in *Drosophila* and mice.

Studies of *parkin* null *Drosophila* have revealed a range of abnormalities. Mutants have reduced longevity [52, 54, 159] and exhibit a slight developmental delay [52]. Pupal lethality was also observed [54]. Males are sterile [52, 54] due to the defects in late spermatogenesis, which may be associated with disrupted integrity of a specialized mitochondrial formation, nebenkern [52]. Mutant animals were also more susceptible to stressors such as paraquat or cold temperature [54]. The mutants have “downturned wing” phenotype and defective flight and climbing ability [52, 54, 159]. Consistently, disruption of muscle integrity and increased apoptosis were detected in the major flight muscles, i.e., the indirect flight muscles (IFM) [52, 54]. Moreover, IFM had severe mitochondrial pathology manifested as swollen mitochondria with disrupted cristae [52, 54]. Strikingly, mitochondrial pathology, muscle degeneration, and climbing and posture defects could be rescued by either total or muscle-specific knockdown of *Marf* (homologue of mitofusins) or *Opa1* and overexpression of *Drp1* [50, 53]. Some studies did not detect the age-dependent loss of DA neurons in the dorsomedial clusters of *parkin*-null *Drosophila* [52, 54], whereas a single study reported severe neuronal loss and shrunken morphology of DA neurons in *parkin* mutant flies [159]. Importantly, if the transgene was selectively expressed in DA neurons, neuronal loss was specifically observed in the PPL1 cluster of *Drosophila* [160, 161]. Furthermore, expression of the *parkin* R275W mutant in *Drosophila* brain, and not the G328E variant, resulted in loss of DA neurons accompanied by climbing defects [161]. Muscle-specific expression of *parkin* R275W in flies also led to certain mitochondrial abnormalities (e.g., disorganized cristae and degenerated mitochondrial membranes) [161]. Another study used *parkin* mutant variants, T240R and Q311X, expressed in DA and 5-HT neurons and reported an age-dependent decline in DA neuron counts in the dorsomedial and dorsolateral clusters, in addition to the defective climbing ability and great difficulty in postural control and coordination [162].

*Parkin* deficiency in mice is not manifested as severe phenotypes observed in fruit flies. A brief summary of mouse models of Parkin deficiency achieved through exon 3 deletion is given in Table 3. Generally, *parkin* mutant mice are viable and fertile [163, 164]. They exhibit normal general appearance, behaviour [163] and longevity [165, 166] and have normal brain morphology [163, 164]. Moreover, loss of *parkin* does not enhance the vulnerability of striatal DA neurons to MPTP toxicity [167]. Surprisingly, no loss of DA neurons in the SN [163, 164, 167, 168], striatum [164] or LC [167] has been detected in the models with exon 3 deletion at 18–24 months of age. Conversely, loss of DA neurons in LC has been observed in *parkin*-deficient mice created via deletion of exon 7. The striatal levels of DA and its major metabolites are mainly unchanged [163, 165], although a single study reported subtle changes in the ratio of DA metabolites [167]. No severe motor impairments were observed, as estimated by the rotarod test [163, 167]. The animals exhibit some non-motor abnormalities, including deficits in the working memory [164], impaired exploratory behaviour [164, 167, 169] and increased anxiety [167]. Alterations of synaptic plasticity in the hippocampal [164, 169, 170] and striatal slices [171] of *parkin*-deficient mice have also been reported, albeit with inconsistent results. However, a study in an exon 2-deletion model detected intact SN and no behavioural defects, including exploratory behaviour, depression-related behaviour, and spatial learning and memory [165]. Analysis of the striatal mitochondria of *parkin*-mutant mice revealed that they did not have apparent swelling, cristae disruption or changes in size and number of mitochondria; however, the mitochondrial respiratory capacity was decreased [172].

**Table 3:** Parkin deficiency in mice with deletion of exon 3

Unchanged parameters	Observed changes
Viability [163, 164] Fertility [163, 164] Normal body mass [164, 167] Normal general appearance [163, 164] Normal brain morphology [163, 164, 167] No brain inclusions [163] No DA neuron loss in SN [163, 164, 167], striatum [164], and locus coeruleus [167] Similar levels of DA, DOPAC and HVA in striatum [163] Similar DA uptake in striatum [163, 171] Similar D1 and D2 receptor binding in striatum [163, 171] No muscle degeneration [164] Normal rotarod performance [163, 167] Normal long-term potentiation in hippocampus [164, 171]	Increased extracellular DA concentration in striatum [163] Increased endogenous DA level in limbic region [164] Reduced levels of DAT and VMAT2 in striatum [164] Reduced synaptic excitability of medium-sized spiny striatal neurons [163] Decreased DA release [171] vs increased DA release in striatum [169] Decreased long-term potentiation in hippocampus [169] Slightly increased paired-pulse facilitation in hippocampus [164] Impaired long-term depression and long-term potentiation in striatum [171] Worse beam traversal task performance [163] Reduced exploratory behaviour [164, 167, 169] Moderate impairment of spatial learning [167] Increased anxiety (light / dark transition test) [167] Decreased recognition index (object location task) [169] Impaired spatial recognition memory (modified Y-maze task) [169]

*DA *dopamine; *DOPAC* 3,4-dihydroxyphenylacetic acid; *HVA *homovanillic acid

Mice expressing *parkin* Q311X in DA neurons exhibit age-dependent hypokinetic motor deficits and loss of DA neurons in SN at 16 months of age [173].

Neuronal degeneration in SN has been observed in *parkin* conditional knockout mice at 9 months of age, in combination with clumping of nuclear chromatin and pyknotic nuclei in SN and mitochondrial alterations in the ventral midbrain neurons (decrease in size and number with abnormal cristae) [73].

The loss of DA neurons in SN or LC was not detected in triple knockout mice, where *parkin* was knocked out along with other two genes mutated in PD, *PINK1* and *DJ-1* [174]. Similar results have been obtained in another triple *parkin*/*DJ-1*/*Gpx1* knockout mouse model [175]. Surprisingly, the *parkin*/*DJ-1*/*Gpx1* and *parkin*/*DJ-1* knockout mice have elevated levels of DA and serotonin in the striatum and perform rotarod task better than the wild-type mice [175].

*Parkin* knockout in MitoPark mice with DA neuron-specific knockout of Tfam (mitochondrial transcription factor A) had no effect on the phenotype or neuronal loss in the SN [176]. Alternatively, Parkin-KO Mutator mice with accelerated generation of mtDNA mutations developed degeneration of DA neurons in the SN and ventral tegmental area at 12 months of age, along with a significant defect in the enzymatic activity of complexes I and III of striatal mitochondria [168]. Similarly, in the PD-mito-PstI mice, where mtDNA undergoes double-strand breaks only in DA neurons, knockout of *parkin* accelerated the loss of DA neurons in SN to appear at 4 months of age [177].

Thus, although Parkin-deficient mice do not fully recapitulate the symptoms of PD patients or the phenotype observed in *Drosophila*, they display a range of pathological alterations; in combination with additional stress that mimics potential environmental damage (e.g., mtDNA mutations), these mice represent a sufficient model for studies of neurodegenerative disorders.

## Conclusions

Pathogenic mutations of the *parkin* gene disrupt the protein at variable degrees and affect its proper function. Considering that Parkin is a widely expressed E3 ubiquitin ligase involved in numerous cellular processes, it is not surprising that the loss-of-function of Parkin can lead to disturbances in functioning of the cell, which in turn result in global pathology. Moreover, Parkin relation to mitochondria may be the link to the mechanisms behind mitochondrial dysfunction in PD.

Mitochondrial dysfunction is one of the critical factors in the development and progression of PD. Moreover, accumulating data suggest that the imbalance in mitochondrial quality control (especially, mitochondrial biogenesis and mitophagy) has deteriorating consequences for the cell, which ultimately lead to the neuronal damage observed in PD. Elucidating the roles of PD-associated genes such as *parkin* can advance the understanding of the importance of mitochondrial integrity and the pathophysiology of PD.

The broad engagement of Parkin in the cellular processes revolving around mitochondria makes it an attractive therapeutic target for intervention, which raises a question of whether stimulation of normal activity of Parkin can potentially ameliorate the dysfunctions observed in PD. Further studies are needed to explore this possibility and to expand our understanding of Parkin functions in health and pathology.

## Data Availability

Not applicable.

## Abbreviations

CCCP: Carbonyl cyanide 3-chlorophenylhydrazone
DA: Dopamine
DOPAC: 3,4-Dihydroxyphenylacetic acid
Drp1: Dynamin-1-like protein
ER: Endoplasmic reticulum
HECT: Homologous-to-the-E6-AP-Carboxyl-Terminus
HVA: Homovanillic acid
IBR: In-Between-Domain
IFM: Indirect flight muscles
LC: Locus coeruleus
Mff: Mitochondrial fission factor
Mfn: Mitofusin
MIM: Mitochondrial inner membrane
Miro: Mitochondrial Rho GTPase
MITOL: Mitochondrial ubiquitin ligase
MOM: Mitochondrial outer membrane
MPTP: 1-methyl-4-phenyl-1,2,3,6-tetrahydropyridine
mTOR: Mammalian target of rapamycin
MTS: Mitochondria targeting sequence
NEMO: NF-kappa-B essential modulator
Opa1: Optic atrophy protein 1
PARIS: Parkin-interacting substrate
PGC1α: Peroxisome-proliferator-activated receptor gamma coactivator 1-alpha
PINK1: PTEN-induced putative kinase protein 1
PD: Parkinson’s disease
PPL1: Protocerebral posterior lateral 1
REP: Repressor Element of Parkin
RING: Really-Interesting-New-Gene
SN: Substantia nigra
TRAK: Trafficking kinesin-binding protein
UbL: Ubiquitin-like domain
VPS35: Vacuolar protein sorting-associated protein 35
